# Knockdown GTSE1 enhances radiosensitivity in non–small‐cell lung cancer through DNA damage repair pathway

**DOI:** 10.1111/jcmm.15165

**Published:** 2020-03-22

**Authors:** Xiao Lei, Lehui Du, Pei Zhang, Na Ma, Yanjie Liang, Yanan Han, Baolin Qu

**Affiliations:** ^1^ Department of Radiation Oncology The First Medical Center of Chinese PLA General Hospital Beijing China

**Keywords:** DNA damage, GTSE1, NSCLC, radiosensitize

## Abstract

Radiotherapy is an important strategy for NSCLC. However, although a variety of comprehensive radiotherapy‐based treatments have dominated the treatment of NSCLC, it cannot be avoided to overcome the growing radioresistance during radiotherapy. The purpose of this study was to elucidate the radiosensitizing effects of NSCLC via knockdown GTSE1 expression and its mechanism. Experiments were performed by using multiple NSCLC cells such as A549, H460 and H1299. Firstly, we found GTSE1 conferred to radioresistance via clonogenic assay and apoptosis assay. Then, we detected the level of DNA damage through comet assay and γH2AX foci, which we could clearly observe knockdown GTSE1 enhance DNA damage after IR. Furthermore, through using laser assay and detecting DNA damage repair early protein expression, we found radiation could induce GTSE1 recruited to DSB site and initiate DNA damage response. Our finding demonstrated that knockdown GTSE1 enhances radiosensitivity in NSCLC through DNA damage repair pathway. This novel observation may have therapeutic implications to improve therapeutic efficacy of radiation.

## 1 INTRODUCTION

Lung cancer is the malignant tumour with the highest prevalence and mortality all over the world, accounting for 30%‐40% in China and other developing countries.^1^, ^2^ According to pathological characteristics, lung cancer can be divided into non–small‐cell lung cancer (NSCLC) and small‐cell lung cancer (SCLC). Non–small‐cell lung cancer includes squamous cell carcinoma, adenocarcinoma and large cell carcinoma, accounting for about 80% of all lung cancers.^3^ At present, the main treatment methods for lung cancer include surgery, radiation therapy, chemotherapy and immunotherapy.^4^, ^5^ Among them, radiotherapy is an important method for local treatment of NSCLC.^6^ However, although a variety of comprehensive radiotherapy‐based treatments have dominated the treatment of NSCLC, it cannot be avoided to overcome the growing radioresistance during radiotherapy.^7^, ^8^ The current research involving radiosensitization mainly falls into the following fields: tumour cell hypoxia tolerance, DNA damage repair, apoptosis, cell cycle disorders and antiangiogenic drugs, etc^9^, ^10^, ^11^, ^12^ However, most of these drugs are in the study process and cannot be applied to clinic for the normal tissue toxicity.^13^

G2 and S phase‐expressed 1 (GTSE1) is a cell cycle‐related protein encoded by the human gtse1 gene, which mainly regulates G1/S cell cycle transition.^14^ Recent years' research shows that GTSE1 is associated with cancer cell survival, metastatic behaviour and chemoresistance.^15^ It has been proved that GTSE1 was related to multiple drug resistance including cisplatin, 5‐fluorouracil and adriamycin.^16^, ^17^ As the role of GTSE1 in tumour getting more and more attention, GTSE1 study involved a variety of tumours including HCC, gastric cancer and breast cancer, except lung cancer.^17^, ^18^, ^19^

Previous studies showed that GTSE1 could shuttle between the cytoplasm and nucleus as a negative regulator of p53. Furthermore, GTSE1 was closely related to DNA damage repair. After DNA damage, GTSE1 accumulated in the nucleus, interacted with p53 and shuttled it out of the nucleus to promote its down‐regulation and recovery,^19^ but the response of GTSE1 to ionizing radiation remains uncovered. In our study, we found that GTSE1 conferred to radioresistance in NSCLC through DNA damage repair pathway. Moreover, we showed that GTSE1 could be recruited to DNA damage repair sites directly after irradiation. These data provide new idea to enhance radiosensitivity in non–small‐cell lung cancer.

## 2 MATERIALS AND METHODS

### 2.1 Cell lines and cell culture

A549, H460 and H1299 were obtained from ATCC (USA). All cell lines were maintained in DMEM with 10% foetal bovine serum at 37°C in a 5% CO2 humidified chamber.

### 2.2 Irradiation

The ^60^Co γ‐rays in Radiation Center (Faculty of Naval Medicine, Second Military Medical University) were used for the irradiation exposure. Cells were treated with 2, 4, 8 Gy of γ‐rays irradiation at a dose rate of 1 Gy/min.

### 2.3 Laser radiation

A 365‐nm pulsed nitrogen laser (Spectra‐Physics) was applied to generate DSBs in a defined area of the nucleus in cells. At 30 minutes after laser radiation, cells were fixed and subjected to immunofluorescence.

### 2.4 siRNA, plasmid and transfections

siRNA against hGTSE1 used was purchased from Thermo Fisher (Catalog # AM16708). Full‐length GTSE1 was constructed by Biolink biotechnology (Shanghai) Co, Ltd. Transfect the plasmid or siRNA with Lipofectamine 3000 (Invitrogen) according to the manufacturer's instructions. Cells were used for further experiments at different times after transfection. The WT and IR groups were all used scrambled siRNA as comparison to siRNA group.

### 2.5 Clonogenic assay

The survival rate of clone formation was used to assess the potential of cell proliferation. The calculated number of cells was seeded in 6‐well plates, irradiated with 0, 2, 4, 8 Gy. After 10 days of incubation, the plates were fixed with paraformaldehyde and stained with 1% methylene blue. Thirty minutes later, wash the plates with PBS and count the clone formation.

### 2.6 Apoptosis assay

Twenty‐four hours after irradiation, cells were stained with an apoptotic detection kit (Invitrogen) with dual staining of Annexin V‐FITC and propidium iodide (PI). Apoptosis was measured and analysed by flow cytometry. (Beckman Cytoflex) according to the manufacturer's instructions.

### 2.7 Comet assay

Neutral comet assay was applied to detect the DNA double‐strand breaks of A549 cells after radiation. The cells were trypsinized at 0 hour, 4 hours, 8 hours after radiation and used comet assay Kit (Trevigen Inc, Gaithersburg, MD) following the manufacturer's protocol, the above 0 hour meant no radiation exposed.

### 2.8 Western blot analysis

At 0 hour, 0.5 hour, 8 hours after irradiation, the proteins from A549 cells were obtained by using ProtecJETTM Mammalian Cell Lysis Reagent (Fermentas, Vilnius, Baltic, Lithuania) according to manufacturer's protocol, then analysed by Western blotting to detect GTSE1 (Abcam, US; 1:1000), p‐DNA‐PKcs (Abcam, US; 1:1000), p‐ATM (Abcam, US; 1:1000), p‐chk1(Abcam, US; 1:1000) and γH2AX(Abcam, US; 1:1000).The secondary antibody (1:5000) was purchased from Cell Signaling Technology, the above 0 hour meant no radiation exposed.

### 2.9 Immunofluorescence analysis

We used immunofluorescence assay to detect the location of GTSE1 and γH2AX foci. Cells were seeded on glass coverslips in a 3 cm petri dish. After washing with PBS, cells were fixed in 3% paraformaldehyde and permeabilized in 0.1% Triton X‐100 in PBS. Then, cells stained with GTSE1 or γH2AX primary antibody (Abcam, US; 1:300) and stained with the secondary antibody (1:1000) at next step. The image was obtained using an Olympus BX60 fluorescent microscope (Olympus America Inc).

### 2.10 Statistical analysis

Data were expressed as mean ± standard error of the mean (SEM) of each experiment. Differences between groups were tested using one‐way analysis of variance. Two groups of comparisons were performed using Student's *t* test for independent samples. *P* < .05 was considered significant. All experiments were repeated at least three independent times. [Correction Statement: Correction added on 08 April 2020 after first online publication: In the Materials and Methods, sub‐sections 2.8 to 2.10 have been renumbered in this version.]

## 3 RESULTS

### 3.1 Knockdown GTSE1 expression by siRNA inhibits the proliferation and promotes apoptosis of NSCLC cells after IR

**Figure 1:**
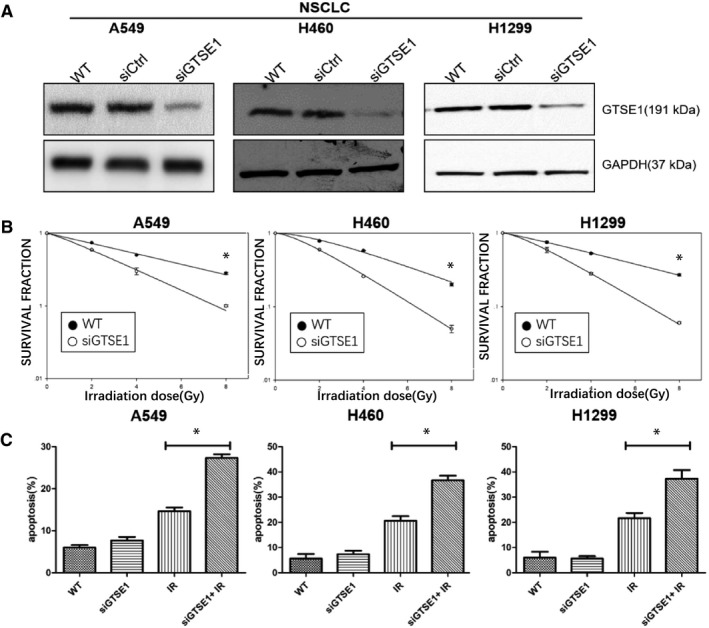
Knockdown GTSE1 expression by siRNA inhibits the proliferation and promotes apoptosis of NSCLC cells after IR. A, GTSE1 expression level was determined by Western bolt assay, GAPDH served as a loading control. B, A549, H460, H1299 and their knockdown GTSE1 cell lines were analysed for their colony‐forming ability against IR. C, A549, H460, H1299 and their knockdown GTSE1 cell lines were analysed for apoptosis by flow cytometric analysis against IR

It has been proved that GTSE1 high expression was related to chemoresistance of multiple cancers. To determine whether GTSE1 participates in radioresistance in NSCLC, firstly we used GTSE1 siRNA to knockdown GTSE1 expression in multiple NSCLC cells (Figure 1A). Then, we used lung cancer cells A549, H1299 and H460 for the next experiments, and our data showed that knockdown GTSE1 expression significantly sensitized these cells to IR (Figure 1B). Furthermore, the apoptosis assay showed that knockdown GTSE1 expression significantly promotes apoptosis of NSCLC cells after IR. (Figure 1C).

### 3.2 GTSE1 overexpressing in GTSE1 knockdown cells rescues the radiosensitive effect after IR

**Figure 2:**
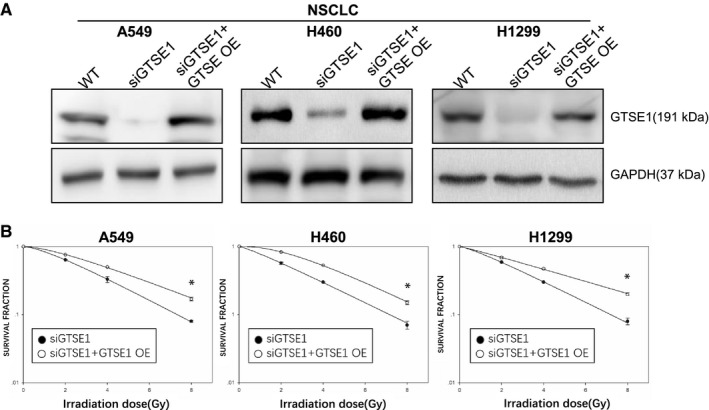
GTSE1 overexpressing in GTSE1 knockdown cells rescues the radiosensitive effect after IR. A, GTSE1 expression level was determined by Western bolt assay, GAPDH served as a loading control. B, knockdown GTSE1 cell lines and their GTSE1 rescue cell lines were analysed for their colony‐forming ability against IR

To fully confirm the role of GTSE1 in NSCLC after IR, we used GTSE1 plasmid to overexpress GTSE1 in GTSE1 knockdown cells. The Western blot result showed in Figure 2A indicated that we had successfully constructed the cell model. Then, we applied these cell model to clonogenic assay, the figure showed GTSE1 overexpressing in GTSE1 knockdown cells successfully rescued the radiosensitive effect in previous experiment after IR (Figure 2B).

### 3.3 Knockdown GTSE1 expression enhances DNA damage of NSCLC cells after IR

**Figure 3:**
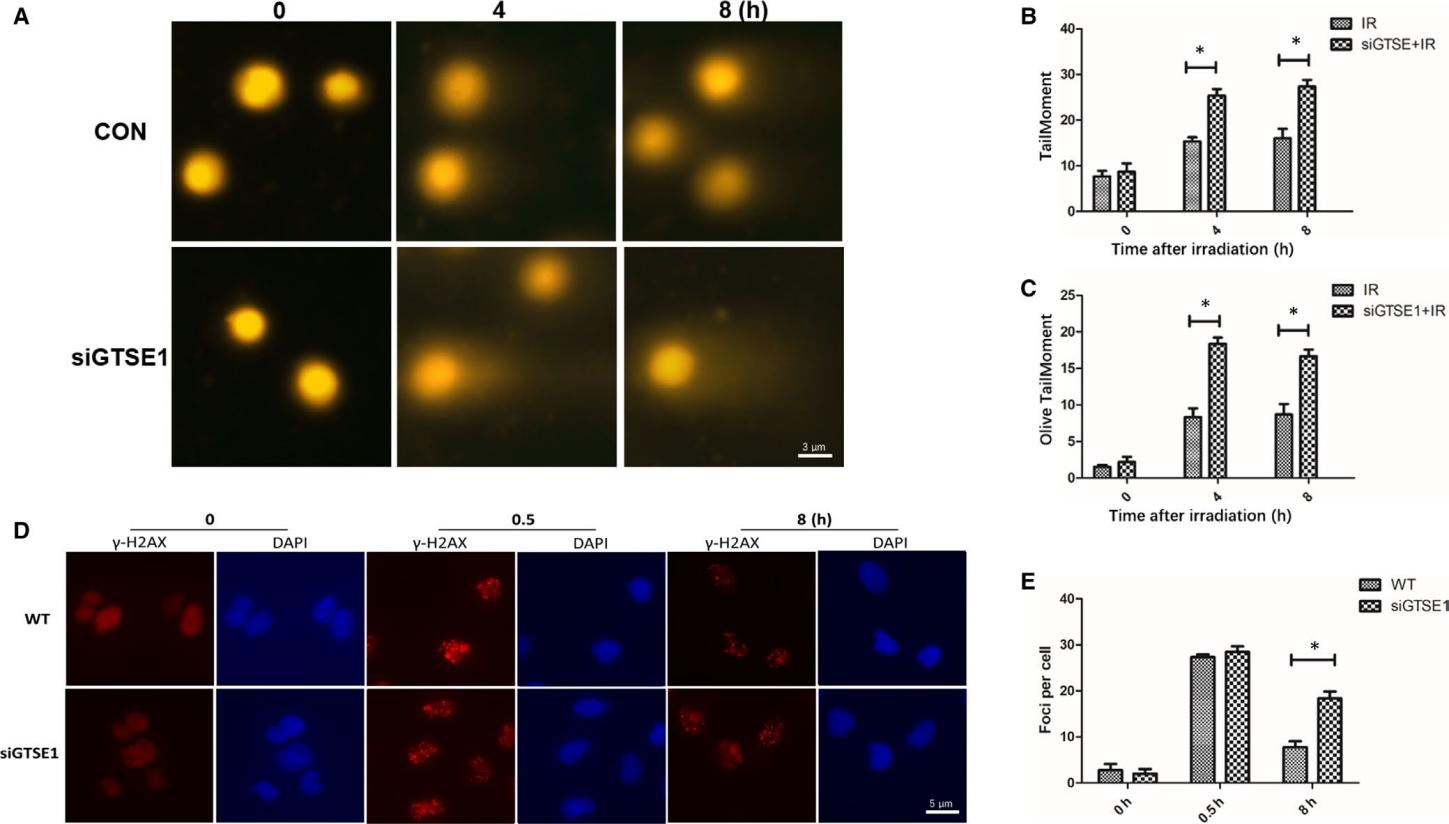
Knockdown GTSE1 expression enhances DNA damage of NSCLC cells after IR. A, Comet assay was utilized to detect the level of DNA damage after IR at different time‐points. n = 3 independent experiments. Quantification in B and C; data represent mean ± SEM. D, A549 and GTSE1 knockdown A549 cells were immunofluorescent stained against γH2AX (red) and DAPI (blue) after IR. E, The γH2AX foci number per cell was calculated among A549 and GTSE1 knockdown A549 cells after IR

To further observe the effect of GTSE1 in lung cancer after IR, the level of DNA damage was assessed via comet assay. As showed in Figure 3A‐C, knockdown GTSE1 expression by siRNA promotes comet formation, thus indicating the radioresistance role of GTSE1 in NSCLC via DNA damage. Then, we used γH2AX assay and found that knockdown GTSE1 significantly impaired DNA repair in response to IR (Figure 3D,E), which meant that GTSE1 might enhance radiosensitivity in NSCLC through DNA damage repair pathway.

### 3.4 Radiation induces GTSE1 recruited to DSB site and initiates DNA damage response

**Figure 4:**
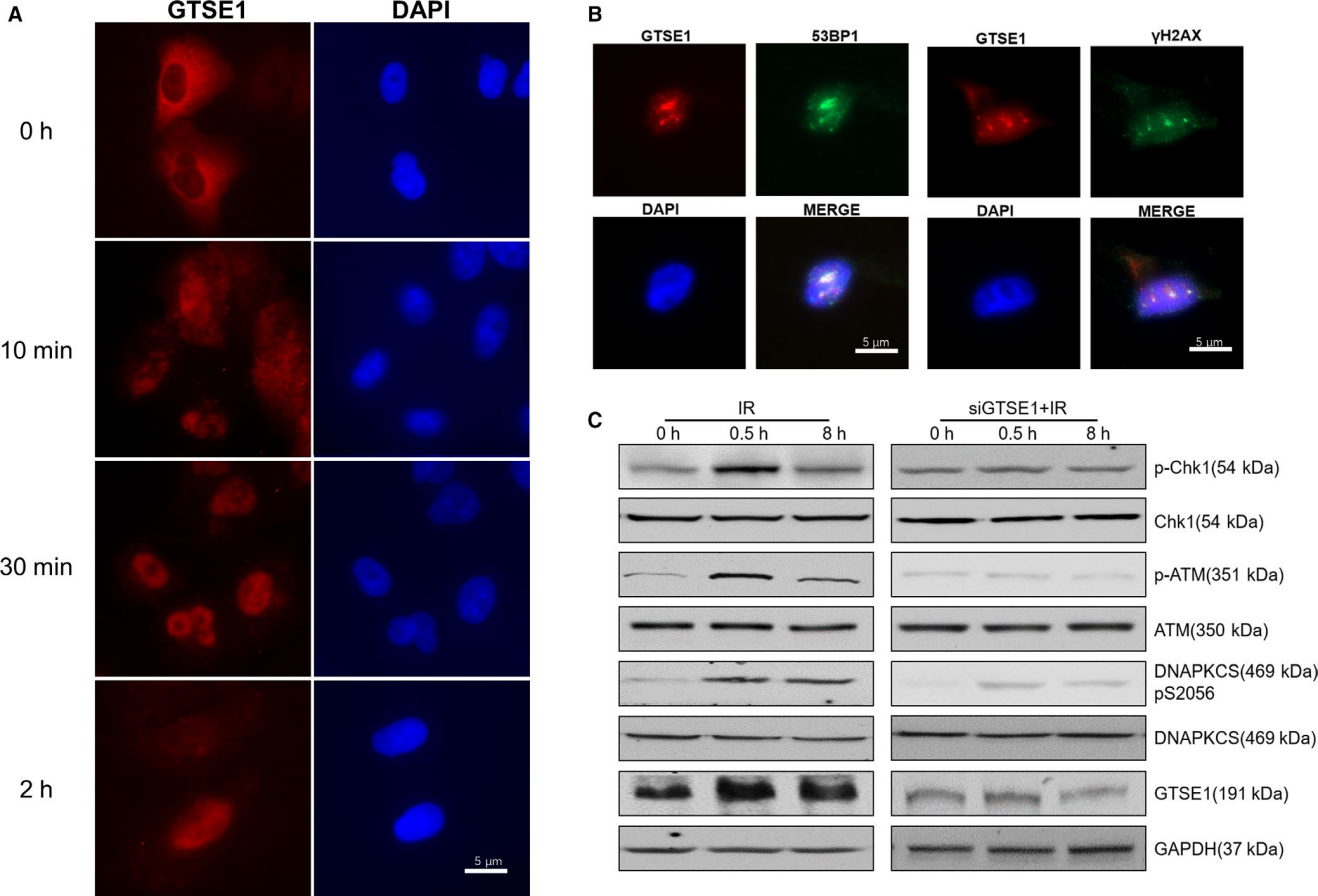
Radiation induces GTSE1 recruited to DSB site and initiates DNA damage response. A, Immunofluorescence of GTSE1 and DAPI in A549 cells after IR. B, immunofluorescence of GTSE1 and 53BP1/γH2AX at DNA damage site following laser microirradiation at 30 min time‐point. C, A549 and GTSE1 knockdown A549 cells exposed to IR harvested at the indicated time‐points, Whole cell lysates were analysed with indicated antibodies

To invest how GTSE1 participates in DNA damage repair pathway, we research the location of GTSE1 after IR. By using immunofluorescence staining and laser assay, we found that radiation induced GTSE1 nuclear translocation rapidly (Figure 4A); moreover, we surprisingly found that GTSE1 could be recruited to DSB site after radiation (Figure 4B), which means GTSE1 might participate in DNA damage repair directly after radiation. Then, we detected the phosphorylation of DNA‐PKcs, ATM and p‐Chk1 etc, which were critical for initiating DNA damage repair, we found that knockdown GTSE1 inhibited the phosphorylation of DNA damage repair proteins (Figure 4C).

## 4 DISCUSSION

In our study, we disclosed the effect of GTSE1 in NSCLC after IR. We utilized three different NSCLC cells to observe the role of GTSE1 through multiple experiments, such as clonogenic assay and apoptosis assay. Besides, we found GTSE1 was closely associated with DNA damage through comet assay. This brought us great interest so that we used immunofluorescence and Western blot analysis to detect the relationship between GTSE1 and DNA damage repair after IR. Then, we revealed that GTSE1 could participate in DNA damage repair pathway, activate DNA damage repair early proteins, so as to confer radioresistance in NSCLC. Our data provide novel insight into targeting GTSE1 in radiosensitization of lung cancer.

### 4.1 Knockdown GTSE1 radiosensitize NSCLC

G2 and S phase‐expressed 1 (GTSE1) was a cell cycle‐related protein encoded by the human gtse1 gene, recent research showed that GTSE1 was associated with cancer cell survival, metastatic behaviour and chemoresistance.^15^ Besides, GTSE1 study involved a variety of tumours including HCC, gastric cancer and breast cancer, except lung cancer.^20^, ^21^

In our study, we found that knockdown GTSE1 expression in NSCLC cells by siRNA significantly inhibited the proliferation and promoted apoptosis after IR. Previous studies showed that GTSE1 could shuttle between the cytoplasm and nucleus as a negative regulator of p53.^19^ To figure out the role of GTSE1 in NSCLC after IR, we detected DNA damage level after IR. Clearly, we found knockdown GTSE1 expression could impair the DNA damage repair process so that DNA damage could not be repaired, which might be associated with GTSE1 radioresistance effect.

### 4.2 Radiation induces GTSE1 recruited to DSB site and initiates DNA damage response

Although we clearly saw the GTSE1 radioresistance effect in NSCLC, the underlying mechanism was largely unknown. Previous study showed that GTSE1 was mainly located in the cytoplasm and was associated with the activity of cytoplasm tubulin and microtubules during mitosis.^22^ After DNA damage, GTSE1 accumulated in the nucleus, interacted with p53 and shuttled it out of the nucleus to promote its down‐regulation and recovery.^19^, ^23^ It was widely accepted that IR could induce DNA damage,^24^ so that we used multiple methods to detect whether GTSE1 participates in DNA damage response. We clearly observed nuclear translocation of GTSE1 in response to IR. Furthermore, we utilized laser radiation and found radiation induces GTSE1 recruited to DSB site, which implied GTSE might participate in DDR pathway directly. Then, we found that GTSE1 inhibition impaired the activation of DNA repair pathway, especially in HR pathway, which is the key mechanism for radiation response. The WB results showed that knockdown GTSE1 could inhibit the phosphorylation of ATM and other DDR pathway early proteins, which indicated that GTSE1 conferred to DNA damage repair after IR and NSCLC radioresistance. But the exact protein GTSE1 activated still need to be disclosed.

In summary, our findings firstly showed knockdown GTSE1 could significantly radiosensitive NSCLC cells. We also discovered GTSE1 could be recruited to DSB site and phosphorylate early DNA damage repair proteins, which indicated GTSE1 conferred to DNA damage repair directly. These new findings could help us discover new mechanism to enhance radiosensitivity in non‐small cell lung cancer.

## CONFLICT OF INTEREST

The authors have no conflicts of interest to disclose.

## AUTHORS' CONTRIBUTIONS

Xiao Lei designed the study, did the most experiments and made the manuscript. Lehui Du and Pei Zhang did some experiments and analysed the data. Na Ma and Yanan Han participated in data collection. Yanjie Liang helped experiments' details. Baolin Qu participated in the writing of paper and revision of manuscript. All authors read and approved the final manuscript.

## Data Availability

The data sets are available under reasonable request.
