# Corrigendum

**DOI:** 10.1111/jcmm.16365

**Published:** 2021-03-05

In Lei et al,^1^ the published article contains errors in Figure 4. The correct figure are shown below. The authors confirm all results and conclusions of this article remain unchanged.

**FIGURE 4:**
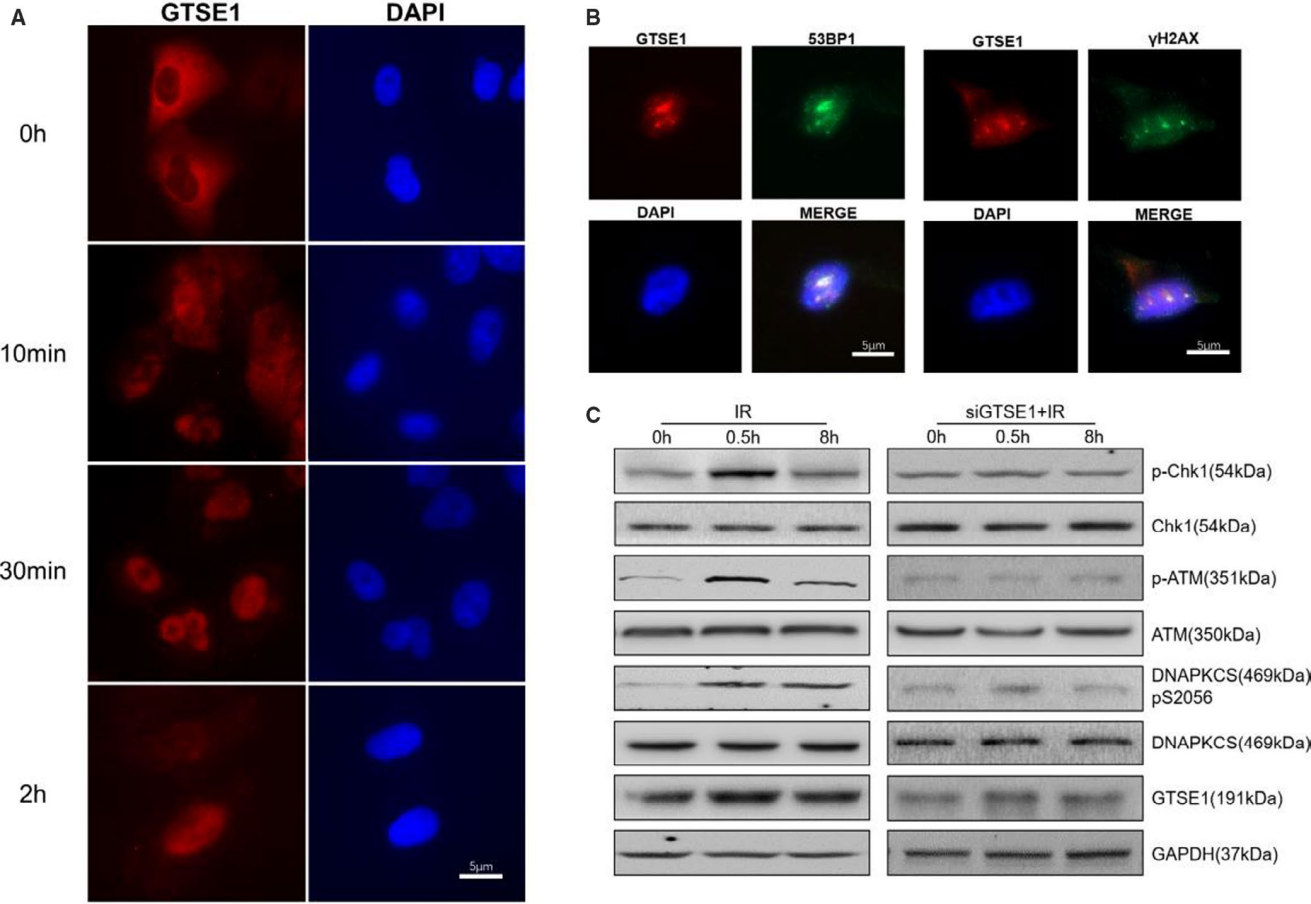
Radiation induces GTSE1 recruited to DSB site and initiates DNA damage response. A, Immunofluorescence of GTSE1 and DAPI in A549 cells after IR. B, immunofluorescence of GTSE1 and 53BP1/γH2AX at DNA damage site following laser microirradiation at 30 min time‐point. C, A549 and GTSE1 knockdown A549 cells exposed to IR harvested at the indicated time‐points, Whole cell lysates were analysed with indicated antibodies
